# Ferroelectric polymer-based flexible and transparent transducers for biomedical ultrasound and photoacoustic imaging^☆^

**DOI:** 10.1016/j.pacs.2026.100899

**Published:** 2026-10-27

**Authors:** Lin Zhang, Senyang Xiao, Shenghao Wu, Jun Xia, Qifa Zhou

**Affiliations:** a Department of Biomedical Engineering, University at Buffalo, The State University of New York, Buffalo, NY 14260, USA; b Alfred E. Mann Department of Biomedical Engineering, University of Southern California, Los Angeles, CA, 90089, USA

**Keywords:** Ferroelectric, Polymers, Transducers, Ultrasound imaging, Photoacoustic imaging, Biomedical applications

## Abstract

Ferroelectric polymers are promising materials for flexible and transparent biomedical ultrasound transducers because of their mechanical compliance, low acoustic impedance, broad bandwidth, optical transparency, and scalable processing. PVDF and its copolymers enable lightweight, conformal interfaces for wearable ultrasound, high-frequency imaging, and photoacoustic/optoacoustic detection, although their relatively modest electromechanical coupling and transmit efficiency remain limitations. This review connects advances in ferroelectric polymer materials with transducer design and biomedical imaging performance. We discuss PVDF, P(VDF-TrFE), relaxor terpolymers, and polymer composites, emphasizing phase and morphotropic phase boundary engineering, molecular modification, and composite strategies. Transducer principles, architectures, fabrication, and performance trade-offs in sensitivity, bandwidth, resolution, penetration depth, flexibility, and stability are critically reviewed. Recent applications in wearable ultrasound, transparent photoacoustic imaging, and ultra-high-frequency and intravascular imaging are highlighted. Finally, challenges and opportunities in material performance, array integration, acoustic coupling, reliability, scalable manufacturing, and multimodal imaging are discussed.

## 1 Introduction

Ferroelectric polymers represent a unique category of electroactive soft materials that bridge the gap between functional inorganic ferroelectrics and mechanically flexible organic systems [1], [2]. Since the discovery of ferroelectricity in poly(vinylidene fluoride) (PVDF) and its copolymers, these materials have been thoroughly investigated for various applications, including flexible sensors [3], actuators [4], mechanical energy harvesting devices [5], [6], capacitive energy storage systems [7], [8], [9], electrocaloric coolers [10], thickness-shear mode resonators [11], acoustic [12] and ultrasound transducers [13]. Their attraction comes from the combination of intrinsic dipolar polarization at the molecular scale with polymeric processability, enabling large-area films, composites with various fillers, multilayer stacks, and mechanically robust structures that are difficult to realize using brittle piezoceramics or single crystals. Continuous advances in polymer chemistry (co-polymers and ter-polymers), crystallization control [14], structure manipulation [15], and electrical poling [16] strategies have steadily improved their electromechanical performance, positioning ferroelectric polymers as enabling materials for next-generation soft electronics.

From this comprehensive viewpoint, ultrasound transducers represent one of the most significant and technically challenging application areas for ferroelectric polymers [17]. Conventional clinical US transducers are dominated by rigid piezoceramics or crystal, such as Lead Zirconate Titanate (PZT), which provide high electromechanical coupling but suffer from large acoustic impedance mismatch with soft tissue, limited bandwidth tailoring, and poor mechanical compliance [18], [19], [20]. Recent advances in conformable and wearable ultrasound electronics have significantly expanded the capability of noninvasive healthcare monitoring beyond traditional rigid ultrasound systems [17], [21], [22]. Innovations in soft piezoelectric materials [23], stretchable interconnects [24], [25], miniaturized electronics [26], and bio-adhesive acoustic interfaces [27] have enabled ultrasound patches that can continuously image and sense deep tissues during daily activities. These systems have demonstrated promising applications in cardiovascular monitoring [28], [29], [30], [31], deep tissue imaging [32], [33], [34], brain monitoring, and multimodal photoacoustic imaging [35], while improving comfort, portability, and long-term signal stability.

The emergence of “soft” ultrasound electronics further highlights the distinctive advantages of ferroelectric polymers over conventional rigid materials. Soft ultrasound systems, which designed to be wearable, skin-conformal, or organ-adaptive, require transducers that can bend, stretch, and maintain intimate acoustic coupling under motion without compromising electromechanical performance [13]. Ferroelectric polymers provide an attractive alternative by combining intrinsic piezoelectric and ferroelectric responses with low acoustic impedance closer to that of biological tissue, naturally promoting broadband operation and efficient acoustic coupling [36], [37], [38]. Furthermore, their extensive mechanical conformal characteristics, elevated strain tolerance, and thin-film configurations facilitate transducer designs that prioritize receive sensitivity, broad bandwidth, and mechanical durability, enabling continuous and user-independent imaging and sensing [4]. These features have motivated sustained interest in polymer-based single-element and array transducers, hybrid multilayer stacks, and piezoelectric micromachined ultrasonic transducers (PMUTs) with polymer architectures for biomedical ultrasound.

Several excellent reviews have previously summarized PVDF-based piezoelectric materials [39], fabrication and preparations [40], fundamental properties [41], flexible piezoelectric sensors [42], [43], energy storage [44], harvesting [45], acoustic and ultrasound transducers [46], [47], and biomedical applications [38]. These studies established important foundations regarding polymer chemistry, ferroelectricity, piezoelectric mechanisms, processing, and broad sensing or actuation applications. However, the rapid development of wearable ultrasound and photoacoustic technologies has created a need for a device-oriented review connecting recent advances in ferroelectric polymer chemistry and phase engineering directly with ultrasound transducer architecture, fabrication, acoustic performance, and biomedical implementation. In contrast to previous reviews centered primarily on materials, general flexible electronics, or individual sensing modalities, the present review emphasizes the complete materials–device–application relationship of ferroelectric polymer-based transparent ultrasound transducers for biomedical imaging. Particular attention is given to recent advances in morcotropic phase boundary (MPB) and molecular engineering, polymer composites, flexible and transparent transducer architectures, practical fabrication and characterization, quantitative acoustic performance, wearable ultrasound/photoacoustic imaging, and emerging multimodal platforms (Fig. 1). This unified framework distinguishes the present review from earlier material- or application-specific reviews by connecting advances in ferroelectric polymer materials with practical wearable acoustic devices and guiding material selection and engineering for specific biomedical imaging applications.

**Fig. 1:**
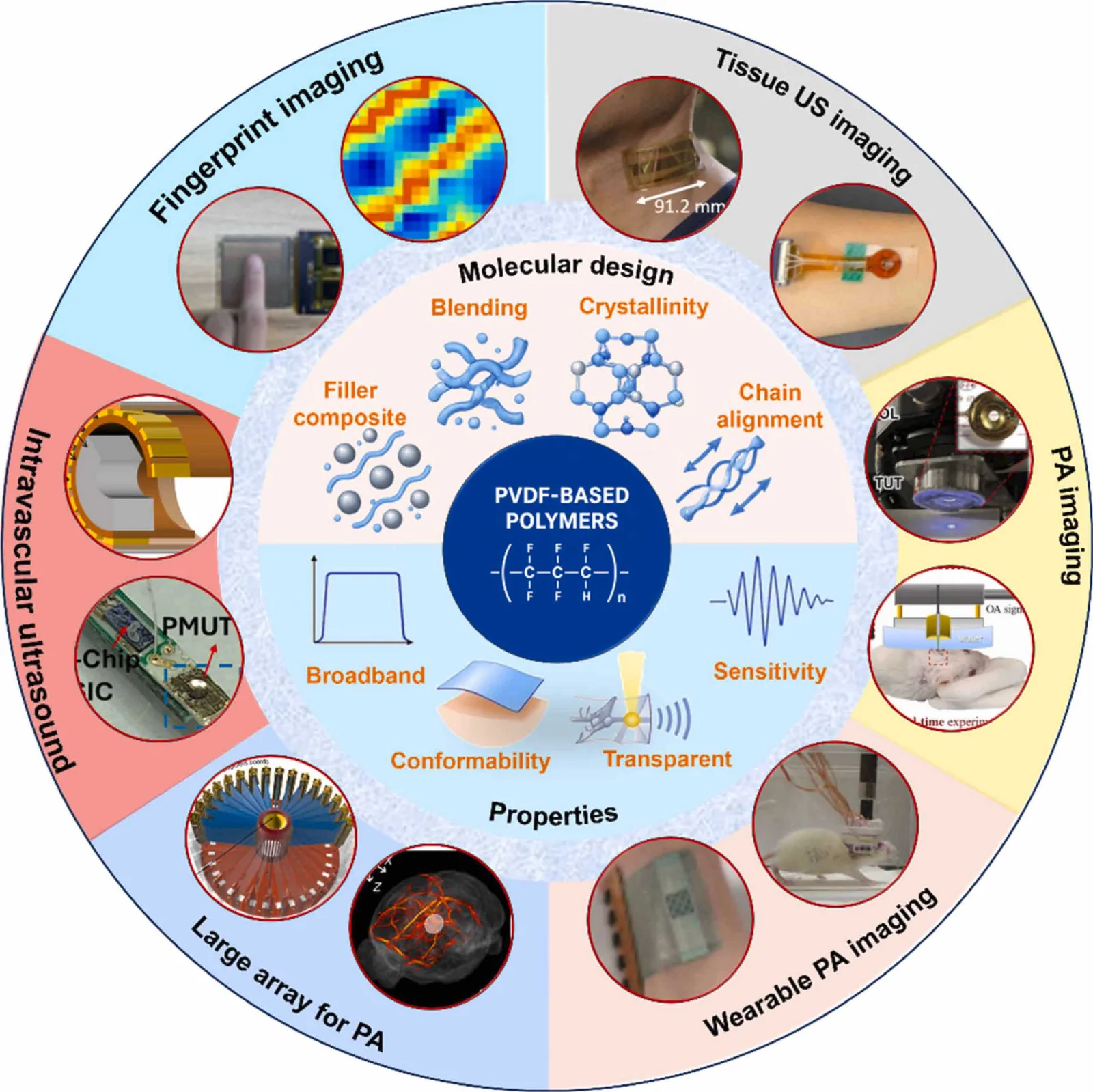
Overview of ferroelectric polymer-based flexible ultrasound transducers, including molecular design, mechanical and acoustic properties, and various biomedical imaging application, including tissue ultrasound imaging [48], [49], fingerprint imaging [50], [51], intravascular ultrasound imaging [52], [53], photoacoustic imaging [54], [55], large area photoacoustic imaging [56], and wearable photoacoustic imaging [57], [58].

## 2 Different types of ferroelectric polymers and composites

### 2.1 Fundamentals of ferroelectric polymer materials

PVDF is a semicrystalline polymer that can crystallize into several conformations, commonly described as α, β, γ, and δ phases [37]. Among them, the all-trans β phase possesses the largest net dipole moment and is therefore particularly important for piezoelectric ultrasound transduction. In unprocessed PVDF, molecular dipoles may be randomly oriented and produce a relatively weak macroscopic piezoelectric response. Mechanical stretching, thermal treatment, electrical poling, epitaxial crystallization, and chemical modification can increase the electroactive phase fraction and/or align molecular dipoles [40].

For ultrasound transducers, material selection cannot be based on $d_{33}$alone. The piezoelectric strain coefficient determines electromechanical conversion, whereas the piezoelectric voltage coefficient g, dielectric permittivity, dielectric loss, electromechanical coupling coefficient, acoustic impedance, mechanical compliance, and thermal stability collectively determine receive sensitivity, transmit efficiency, bandwidth, acoustic matching, and device durability [47]. PVDF generally offers high voltage sensitivity, low acoustic impedance, and excellent flexibility, whereas P(VDF-TrFE) provides more stable ferroelectric ordering and generally stronger piezoelectric response. Relaxor terpolymers offer high dielectric response and electrostrictive strain but can exhibit increased dielectric loss and lower thermal stability. Polymer composites provide another route to enhance electromechanical performance, although filler loading introduces additional mechanical and acoustic trade-offs.

### 2.2 PVDF-based polymers

Ferroelectricity in PVDF-based polymers originates from molecular dipoles associated with the C–F bonds and their alignment in specific crystalline conformations [37]. The fundamental structural concept is the percent of electroactive phases, particularly the β phase (all-trans conformation), which produces the highest spontaneous polarization and piezoelectric response [1], [59]. Processing routes such as mechanical stretching, electrical poling, and epitaxial crystallization are widely used to raise β-phase content and to stabilize dipole alignment. The properties of representative polymers are listed in Table 1. In device-relevant terms, PVDF-family films behave as anisotropic piezoelectric layers whose performance depends not only on intrinsic coefficients (e.g., $d_{33}$, $d_{31}$, $g_{33}$) but also on dielectric permittivity, mechanical compliance, and loss, which together determine voltage sensitivity, bandwidth, and acoustic power when used for actuators [46] or ultrasound applications [60].

**Table 1:** Comparison of ferroelectric polymer materials for ultrasound transducers.

Property	PVDF	P(VDF–TrFE)	PVDF–TrFE–CFE
Ferroelectricity	Weak / processing-dependent	Strong, robust	Relaxor-like ferroelectric
Piezoelectric coefficient d₃₃ (pC N⁻¹)	20–30	25–40	10–25
Voltage coefficient *g*₃₃ (V m N⁻¹)	200–330	150–300	80–200
Relative permittivity εᵣ (1 kHz)	8–12	12–20	30–50
Dielectric loss (tan δ)	Low (≈0.02–0.05)	Moderate (≈0.03–0.06)	Higher (≈0.05–0.10)
Electromechanical coupling (kₜ)	Low–moderate	Moderate	Low–moderate
Acoustic impedance (MRayl)	~3–4	~4–5	~4–5
Curie / ferroelectric transition	No sharp T_c_ (melting ~170 °C)	T_c_ ≈ 100–130 °C	Diffuse transition ~50–80 °C
Thermal stability for US use	≤ 80–100 °C	≤ 100–120 °C	≤ 60–80 °C
Electrostriction	Weak	Moderate	Strong (large strain)
Typical processing	Stretching + high-field poling	Solvent casting annealing + low-field poling	Solvent casting + moderate poling
Poling field	High (80–120 MV m⁻¹)	Lower (50–80 MV m⁻¹)	30–60 MV m⁻¹
Film thickness (typical)	5–50 µm	5–50 µm	5–30 µm
Strengths for ultrasound	High *g*, low loss, broadband receive	Best balance of sensitivity & stability	Large strain, tunable dielectric
Limitations	Difficult phase control	Lower output than ceramics	Higher loss, lower T stability
Typical roles	large-area sensing, moderate-frequency transduction	imaging elements, focused transducers, arrays	actuation-heavy devices; emerging ultrasound stacks
Reference	[15], [47], [61]	[15], [46], [62]	[15], [46]

PVDF (homopolymer) is one of the most widely studied ferroelectric polymers because it combines mechanical flexibility, biocompatibility, chemical stability, and intrinsic piezoelectricity [63]. The piezoelectric response of PVDF mainly originates from its polar β-phase, in which the molecular dipoles are aligned in the all-trans (TTTT) conformation. As shown in Fig. 2, after mechanical stretching and electrical poling, PVDF films can exhibit piezoelectric coefficients ($d_{33}$) typically in the range of −20 pC/N. Although PVDF exhibits inferior electromechanical coupling properties, it retains the benefits of a conventional polymer, including chemical tailorability, functionalization, and diminished stiffness, which enhances flexibility and conformability essential for wearable applications. Moreover, PVDF possesses an acoustic impedance (1.6–6 MRayl) comparable to that of human tissue (1.5 MRayl), thereby obviating the necessity for - matching layers [37], [64].

**Fig. 2:**
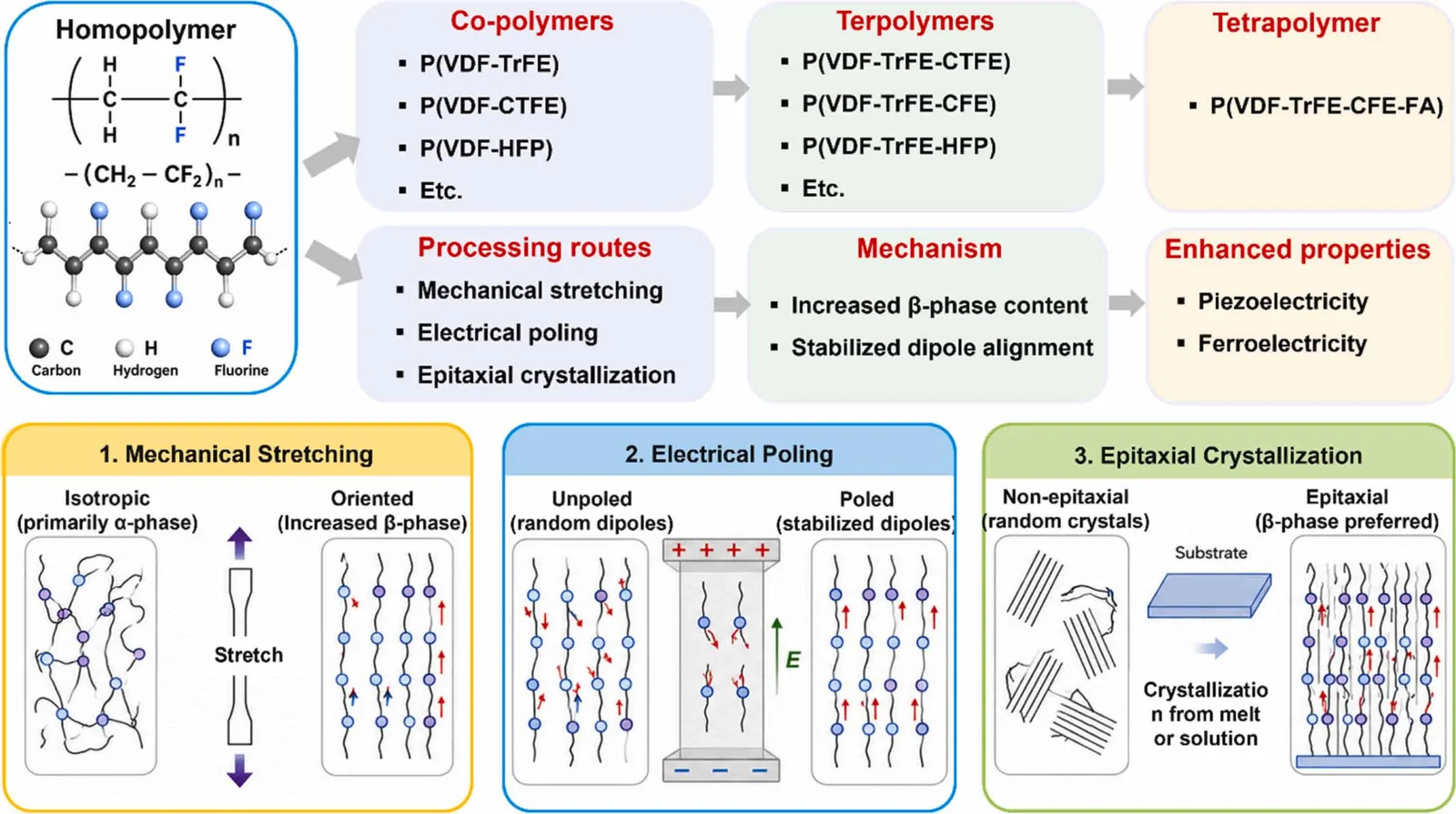
Overview of PVDF-based ferroelectric polymers and representative strategies for enhancing their electroactive phases and electromechanical properties.

A particularly important recent development is the discovery and subsequent engineering of morphotropic phase boundary (MPB)-like behavior in ferroelectric polymers [65]. MPBs are well established in high-performance piezoelectric ceramics, where competition between energetically comparable polar phases facilitates polarization rotation and produces enhanced electromechanical response. PVDF-based copolymers, particularly poly(vinylidene fluoride-trifluoroethylene) [P(VDF-TrFE)], were developed to further enhance ferroelectric, piezoelectric, and energy storage performance [66], [67], [68], [69]. The incorporation of TrFE units stabilizes the polar crystalline phase and increases crystallinity, enabling stronger spontaneous polarization and easier ferroelectric switching without requiring extensive mechanical stretching [12], [70], [71]. As a result, P(VDF-TrFE) typically exhibits greater piezoelectric coefficients than pure PVDF, with reported $d_{33}$ values of −40 pC/N and a $k_{33}$ value of 33%. Liu *et al.* reported a $d_{33}$ of −65 pm/V and $k_{33}$ < 27% for P(VDF-TrFE) copolymers by utilizing the morphotropic phase boundary (MPB) effect in compositions near the MPB [65]. The behavior reminiscent of an MPB in ferroelectric polymers demonstrated that this concept can also be exploited in molecularly engineered polymer systems. Other copolymers such as P(VDF-HFP) and P(VDF-CTFE) have also been explored to tailor dielectric [72], [73], [74], [75], [76], [77] and electroactive properties [66] for flexible electronics. More recent studies have further advanced this strategy through scalable solution processing [78], crosslinking [79], strain-induced domain engineering [70], [80], electric poling [81], and intramolecular modification, producing substantially enhanced piezoelectric responses while retaining the mechanical compliance and processability characteristic of polymers [15], [82], [83]. These developments represent an important transition from conventional β-phase enhancement toward deliberate engineering of competing polar states and nanoscale domain structures [8], [84], [85]. These copolymers exhibit improved processability, enhanced electromechanical stability, and better compatibility with nanofillers and conductive additives, enabling high-performance flexible transducers and multimodal sensing platforms [86], [87]. Importantly, MPB-engineered polymers have already enabled highly sensitive flexible devices for detecting mechanical signals such as pressure and sound, indicating their potential to address the historically limited transmit sensitivity of polymer ultrasound transducers [88], [89]. Incorporating such high-performance polymers into flexible ultrasound arrays, transparent photoacoustic receivers, and wearable acoustic interfaces may therefore provide an important route toward simultaneously improving sensitivity and mechanical conformability.

PVDF-based terpolymers and tetrapolymer, including P(VDF-TrFE-CTFE) [1], P(VDF-TrFE-CFE) [90], [91] and P(VDF-TrFE-CFE-FA) [92], further expand the electromechanical functionality of fluorinated ferroelectric polymers [40]. The introduction of a third bulky monomer disrupts long-range ferroelectric ordering and produces relaxor-ferroelectric behavior, leading to exceptionally large electrostrictive strain and enhanced dielectric response. For example, the terpolymer with a composition of VDF/TrFE/CTFE = 64.5/33.1/2.4 mol% exhibits a *d*_33_ of −55.4 pC/N [90]. Although their direct piezoelectric coefficient may not always exceed optimized copolymers, these terpolymers exhibit outstanding electromechanical deformation, low hysteresis, and high energy density, making them promise for soft actuators, adaptive ultrasound devices, wearable haptics, and energy conversion systems.

### 2.3 Polymer based composites

PVDF-based composites with various fillers have been widely investigated as sensors [11], energy storage devices [93], [94], [95], [96], harvesters [5], and transducers [12]. Compared with pure PVDF polymers, composite can improve performance through several mechanisms. First, ceramic or polar nanofillers can promote β-phase crystallization, increasing the fraction of the ferroelectric phase responsible for piezoelectric activity. Second, the filler-polymer interface can locally concentrate electric field and stress [76], which can improve dipole alignment during poling and increase effective sensitivity. Third, selected fillers can raise dielectric constant and electromechanical response without completely sacrificing flexibility [95]. Fourth, it is better acoustic matching to tissue, which reduces reflection losses at the front face and can simplify matching-layer design. Furthermore, flexible piezoelectric composites with high *d*_33_ and *g*_33_ piezoelectric coefficients (i.e. high figure of merit) at high frequencies should be used for efficient wireless ultrasonic powering. For ultrasound imaging specifically, this means PVDF composites can address one of the main shortcomings of pure polymer transducers: limited transmit/receive sensitivity. Pure PVDF maximizes compliance and broadband reception, while PVDF composites aim for a higher output and sensitivity than neat polymers but lower stiffness and acoustic mismatch than all-ceramic designs.

As a result, composite PVDF transducers can deliver a more favorable balance of sensitivity and bandwidth than pure polymer films. That middle level is increasingly important for high-frequency imaging, wearable patches, and anatomically conformal arrays, where conventional ceramics remain difficult to deploy despite their strong intrinsic piezoelectricity. These mechanisms are reflected experimentally in PVDF with BaTiO_3_ (25 MHz with 156% of BW) [97], Ba_0.8_Sr_0.2_TiO_3_ (39 MHz with 84% of BW) [98], (NaBi)TiO_3_-BaTiO_3_ [99] and Bi_2_O_3_ (14 MHz with 128% of BW) [100] and PMN-PT [101]. Recently, N. Das *et al.* employed P(VDF-TrFE) thin film integrated with a transition metal hydroxide (β-Ni(OH)_2_ nanoplates) and demonstrated its large bandwidth 51.22 MHz with 102.4% of BW and peak-to-peak output voltage of 5.19 V, which promises significantly enhanced ultrasound image resolution [102]. Wang *et al.* reported P(VDF−TrFE) and black phosphorus nanosheets piezoelectric composite with a sensitivity up to 3.2 mV kPa^−1^ and high spatial resolution of 50 μm, displaying the contours and shapes of fingerprints and hairlines [50]. Using the laser graphitization with 3D printing, Movaghgharnezhad and co-workers fabricated flexible porous graphene (LIG) /PVDF-TrFE ultrasound patches for B-mode image of a cyst phantom [49]. The LIG-based ultrasound transducer demonstrates a high signal amplitude of 6.72 V and a signal-to-noise ratio (SNR) of 433, along with a −6 dB bandwidth of 8.86 MHz (37%). In addition, some organic dopants, such as polypyrrole with amino groups [103], [104], can facilitate dipole alignment and enhance piezoelectricity by forming relatively weak hydrogen bonds [105].

Despite these advancements, several issues still prevent PVDF-based composite transducers from being widely used in mainstream ultrasound imaging, such as filler aggregation leading to dielectric loss, stress inhomogeneity, and inconsistent poling. The trade-off between sensitivity, bandwidth, and flexibility is another factor to consider because increasing filler may boost piezoelectric response but also increase stiffness, attenuation, and acoustic impedance. The most promising path for PVDF-based composite transducers in the future is to use them as the active material basis for multimodal, conformable, and application-specific ultrasound systems. Hierarchical composites are one approach that optimizes stress transfer and bandwidth simultaneously by co-engineering a fluoropolymer matrix with nanofillers, porous or textured electrodes, and customized backing/matching materials. Large-area and long-duration monitoring platforms are another trend, and PVDF composites may be particularly useful in situations where comfort, conformability, and ease of manufacturing are crucial but moderate sensitivity is acceptable.

## 3 Design, fabrication, and characterization for flexible ultrasound transducers

### 3.1 Operating principle

Ferroelectric polymer ultrasound transducers rely on the direct and inverse piezoelectric effects [47]. During ultrasound transmission, an alternating electric field applied across a polarized polymer layer produces periodic thickness deformation through the inverse piezoelectric effect, generating acoustic waves. During reception, incident acoustic pressure deforms the polymer and produces electrical charge through the direct piezoelectric effect. A single element therefore can operate as both an ultrasound transmitter and receiver. The acoustic performance is governed by several coupled material and structural parameters [106]. The piezoelectric coefficients determine electromechanical conversion, while dielectric permittivity and loss influence electrical impedance, voltage sensitivity, and power consumption [62]. The low acoustic impedance and mechanical Q-factor of PVDF-family polymers favor broadband operation and short acoustic pulses. The active-layer thickness strongly influences resonance frequency; consequently, thin films are particularly advantageous for high- and ultra-high-frequency transducers. In flexible arrays, element dimensions and pitch additionally determine beam steering, grating lobes, lateral resolution, and imaging field of view.

### 3.2 Design and structure

The design of PVDF-based flexible ultrasound transducers focuses on achieving high electromechanical sensitivity, mechanical flexibility, acoustic matching, and stable conformal interfacing with biological tissues. As shown in Fig. 3, Typical device structures consist of multilayer architectures including a top electrode, PVDF piezoelectric active layer, bottom electrode, flexible substrate layer, and encapsulation layer [49]. Sometimes the backing layer is also attached to improve the bandwidth but sacrifice the flexibility [51], [102]. In single-element transducers, the active PVDF film is generally sandwiched between conductive electrodes to generate and detect ultrasound waves through the direct and inverse piezoelectric effects [49], [50], [107], [108], [109]. For flexible array transducers, multiple patterned electrode channels are introduced to enable beam steering, phased-array imaging, spatial mapping, and multiplexed sensing [57]. Flexible substrates such as polyimide (PI), polyethylene terephthalate (PET), and polydimethylsiloxane (PDMS) are commonly employed to provide mechanical compliance and stretchability [48], [110]. For PMUT design, a thin PVDF piezoelectric membrane integrated with top and bottom electrodes is suspended over a micromachined cavity, allowing membrane vibration for efficient ultrasound transmission and reception [52], [111]. Such micromachined architecture allows miniaturization, low-power operation, and integration with wearable electronics and CMOS circuitry.

**Fig. 3:**
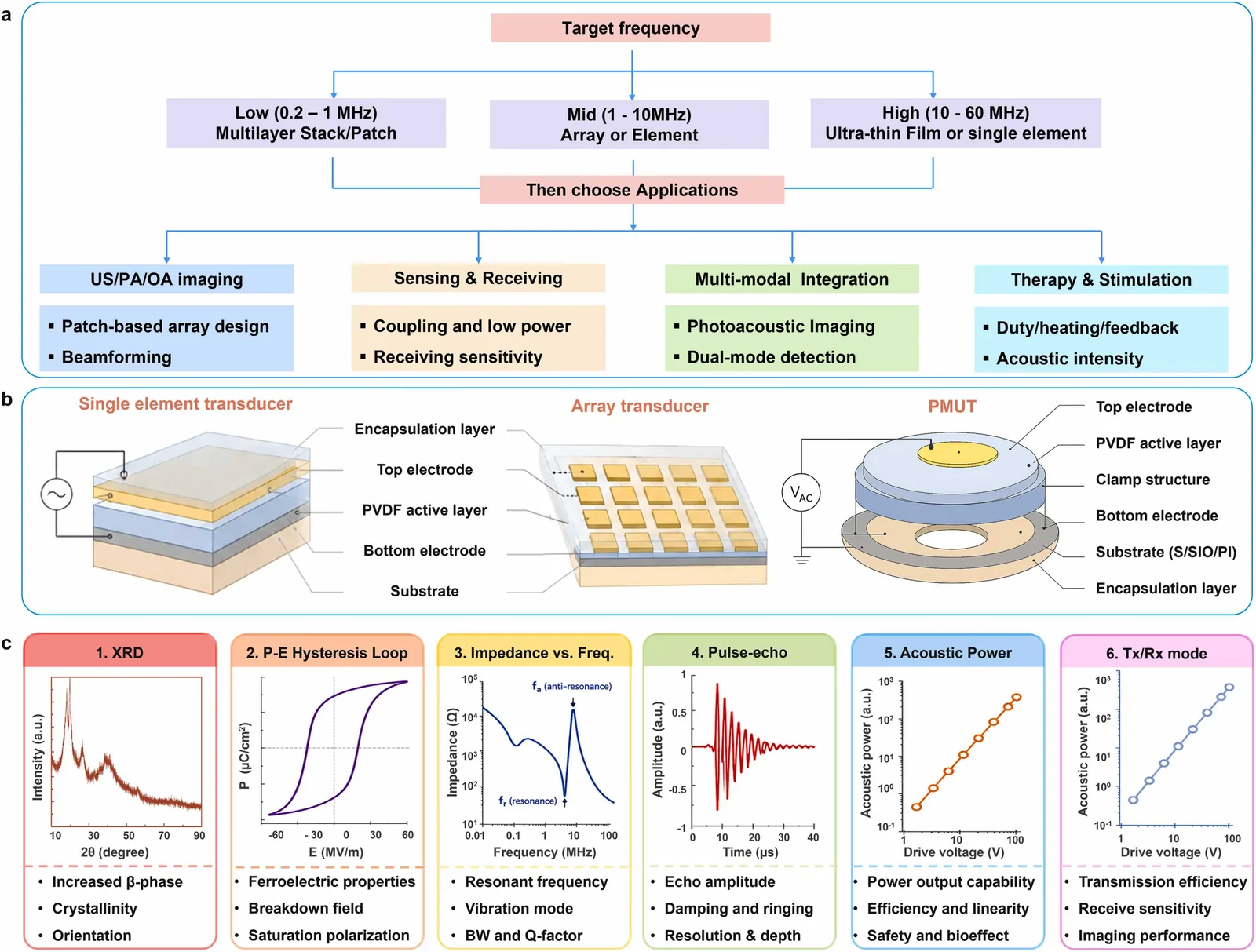
(a) Design, (b) structure, and (c) characteristics of PVDF-based ultrasound transducers.

The piezoelectric performance strongly depends on the β-phase crystalline content and dipole alignment of PVDF polymers. Therefore, device geometry, membrane thickness, acoustic impedance matching, and electromechanical coupling are critical design considerations. Thin PVDF layers typically ranging from several micrometers to tens of micrometers are used to achieve broad bandwidth and high-frequency operation. Electrode materials such as Au, Pt, Al, Ag nanowires, PEDOT:PSS, graphene, and transparent conductive oxides are selected based on conductivity, flexibility, optical transparency, and acoustic attenuation. Encapsulation layers additionally serve as acoustic matching layers to improve transmission efficiency and protect devices from moisture and mechanical damage.

### 3.3 Fabrication and Characterization

Fabrication of PVDF-based flexible ultrasound transducers generally involves polymer film formation, electrode deposition, polarization treatment, microfabrication patterning, and device encapsulation. PVDF and its copolymers or terpolymers can be processed using solution casting [50], spin coating [102], [109], [112], hot embossing [48], electrospinning [113], [114], [115], [116], spray printing [117], melt extrusion, and inkjet printing [49], [118], [119] methods. Among these techniques, solution casting and spin coating are widely used because they allow precise control over film thickness and compatibility with flexible substrates. Electrospinning has also attracted significant interest due to its capability to induce highly aligned nanofibers with enhanced β-phase crystallinity and piezoelectric performance. To enhance piezoelectricity, several post-processing routes are commonly employed, including mechanical stretching, electrical poling, thermal annealing, and epitaxial crystallization. Mechanical stretching transforms nonpolar α-phase PVDF into the polar β-phase by inducing all-trans chain conformations. Electrical poling under a high electric field further aligns dipoles and stabilizes ferroelectric polarization. In PVDF based composite systems, functional fillers such as carbon nanotubes, graphene, ceramics, nanocellulose, and MXenes can additionally promote β-phase nucleation and improve dielectric and electromechanical properties.

Electrode fabrication is typically achieved through sputtering, evaporation, screen printing [119], aerosol printing, or transfer printing techniques. Patterned electrodes are especially important for ultrasound arrays and PMUT architectures. Microfabrication approaches including photolithography, laser ablation, lift-off processing, and reactive ion etching are often utilized to define array elements and interconnects. Encapsulation layers such as PDMS, polyurethane, Ecoflex, and Parylene C are subsequently deposited using spin coating, spray coating, dip coating, or chemical vapor deposition to provide waterproofing, mechanical protection, acoustic matching, and biocompatibility. For PMUT fabrication, additional micromachining processes including cavity etching, sacrificial layer release, and membrane transfer are incorporated to realize suspended piezoelectric diaphragms. As shown in Fig. 3c, ferroelectric and piezoelectric properties are typically evaluated using polarization–electric field (P–E) hysteresis loops, piezoresponse force microscopy (PFM), and piezoelectric coefficient measurements (*d*_33_ or *d*_31_) [119]. Electrical impedance analysis is another key characterization method, allowing identification of resonance and anti-resonance frequencies, electromechanical coupling coefficients, and bandwidth characteristics. Impedance-versus-frequency measurements are especially important for optimizing ultrasound transmission efficiency and imaging sensitivity. Acoustic characterization includes pulse–echo measurements, transmit/receive (TX/RX) testing, hydrophone measurements, and acoustic power analysis. Pulse–echo testing evaluates time-domain ultrasound responses, center frequency, axial resolution, damping behavior, and signal-to-noise ratio. TX/RX measurements demonstrate the ability of the transducer to both generate and detect ultrasound signals for imaging and sensing applications. Acoustic output pressure and power are often quantified using calibrated hydrophones to assess transmission efficiency and therapeutic capability.

### 3.4 Material and architecture selection: performance trade-offs

No single ferroelectric polymer composition or transducer architecture simultaneously maximizes sensitivity, bandwidth, imaging depth, spatial resolution, flexibility, and long-term stability. Instead, device performance reflects coupled trade-offs among material electromechanical properties, acoustic impedance, active-layer thickness, geometry, backing, electrodes, encapsulation, operating frequency and mechanical flexibility.

PVDF homopolymer provides relatively low acoustic impedance, high voltage sensitivity, and broad bandwidth, but a low electromechanical coupling and piezoelectric strain coefficient, limit transmit pressure and consequently constrain penetration depth and pulse-echo sensitivity [17], [63]. P(VDF-TrFE) generally provides stronger and more stable ferroelectric polarization and higher piezoelectric response than unmodified PVDF while retaining substantial flexibility, reported thickness-mode coupling coefficients are approximately 0.27–0.30, compared with approximately 0.15–0.20 for PVDF [47]. It therefore offers a useful compromise for flexible pulse-echo imaging and arrays, although its acoustic output remains lower than that of high-performance ceramics or single crystals. Recent molecular and MPB engineering may further narrow this sensitivity gap [120]. Relaxor terpolymers can exhibit large dielectric/electromechanical responses, making them attractive for actuation and emerging high-sensitivity devices. Their higher dielectric loss and composition-dependent thermal stability, however, require careful consideration for high-frequency and long-duration operation [121], [122]. Polymer composites can increase piezoelectric response and transmit sensitivity through ceramic, nanostructured, or conductive fillers. This enhancement may improve SNR and imaging depth, but increasing filler loading generally increases stiffness, acoustic impedance, attenuation, and dielectric loss and may reduce conformability. Thus, composite optimization requires balancing electromechanical gain against acoustic and mechanical penalties.

Device architecture introduces an additional level of optimization. Thin-film single-element transducers provide simple fabrication, high flexibility, and broad bandwidth but limited spatial encoding. Patterned arrays enable beamforming, electronic focusing, and spatial imaging, but require higher channel density and introduce challenges associated with interconnects, cross-talk, element uniformity, and bending-induced geometric variation. PMUT architectures offer miniaturization, low-power operation, and compatibility with microelectronics, but their flexural-mode behavior and membrane fabrication introduce additional design constraints. Similarly, backing layers can broaden bandwidth and suppress ringing but may increase thickness and reduce mechanical flexibility. Operating frequency further determines the imaging trade-off. Increasing frequency and reducing polymer thickness improve axial resolution but increase tissue attenuation and therefore reduce penetration depth. Lower-frequency devices penetrate deeper tissues but require thicker active layers and generally provide lower spatial resolution. Consequently, high-frequency PVDF devices are particularly suitable for superficial, ophthalmic, intravascular, and microscopic imaging, whereas deeper wearable imaging requires improved transmit efficiency, larger apertures, optimized composites or multilayer structures, and sensitive low-noise electronics.

## 4 Ferroelectric polymer-based transducers for biomedical applications

Ferroelectric polymer-based ultrasound transducers have emerged as promising platforms for biomedical imaging devices due to their flexibility, lightweight nature, biocompatibility, and acoustic compatibility with soft tissues. As illustrated in Fig. 1, these transducers enable a broad range of applications including deep tissue ultrasound imaging and photoacoustic/optoacoustic imaging. In addition, their ability to simultaneously provide imaging, sensing, stimulation, and therapeutic functionalities within soft and skin-conformal systems positions ferroelectric polymer ultrasound devices as key enabling technologies for next-generation personalized healthcare, continuous physiological monitoring, and intelligent bioelectronic medicine.

### 4.1 Wearable and conformal imaging

Wearable ultrasound imaging aims to change ultrasound from a stationary, operator-dependent exam to continuous or repeatable imaging outside the clinic, such as at home, during rehabilitation, or in ambulatory settings [13]. The key technical driver is mechanical conformity, which by matching the curvature and motion of skin (or soft tissue surfaces), a patch can stabilize acoustic coupling and reduce probe-to-probe variability that otherwise dominates image quality and measurement repeatability [123]. Stretchable array architectures as the solution for rigid piezoelectric ceramics, including island-bridge layouts, stretchable encapsulants, and multiplexed readouts, demonstrated how nonplanar surfaces can be imaged with stable contact and reduced geometric artifacts, highlighting why conformal mechanics is not merely ergonomic but directly tied to beamforming quality and repeatability [24], [29].

Unlike rigid piezoelectric ceramics (e.g., PZT, PMN-PT), PVDF films can be fabricated into thin, lightweight, and stretchable transducer arrays that conform intimately to curved and dynamic body surfaces, enabling stable acoustic coupling without bulky fixtures [124]. Their wide bandwidth supports high axial resolution imaging, while their mechanical compliance minimizes motion artifacts during long-term monitoring [4]. Recent work has demonstrated PVDF-based conformal ultrasound patches for applications such as continuous cardiovascular and hemodynamic monitoring [48], finger blood vessels and skin [97], cyst imaging [49], and fingerprint imaging [50], [51]. Neer *et al.* introduced next-generation wearable sensing platforms enabled by pillar structured P(VDF-TrFE) and multimodal signal decoupling, improving accuracy, reliability, and real-time monitoring capabilities (Fig. 4a-e). It and can observe axial lengths of the PSF were 0.23 and 0.92 mm at the −6and −20 dB levels on the 040GSE phantom and clear echoes of the vessel walls. The low acoustic impedance of P(VDF-TrFE) enables a large frequency bandwidth and a high axial resolution without the necessity of matching or backing layers. In another work, using the laser graphitization with 3D printing, Movaghgharnezhad and co-workers fabricated flexible porous graphene (LIG) /PVDF-TrFE ultrasound patches (Fig. 4f-g), which demonstrated a high signal amplitude of 6.72 V, along with a −6 dB bandwidth of 8.86 MHz (37%), achieving sufficient resolution on a cyst phantom [49]. Furthermore, some researchers demonstrated ultrathin, skin-conformal single transducer capable of resolving fine spatial features such as epidermal ridge patterns. In fingerprint imaging (Fig. 4h-n), this requires both high spatial resolution and uniform contact across curved, compliant skin, which these studies achieve through stretchable substrates, dense electrode/pixel integration, and improved electromechanical coupling.

**Fig. 4:**
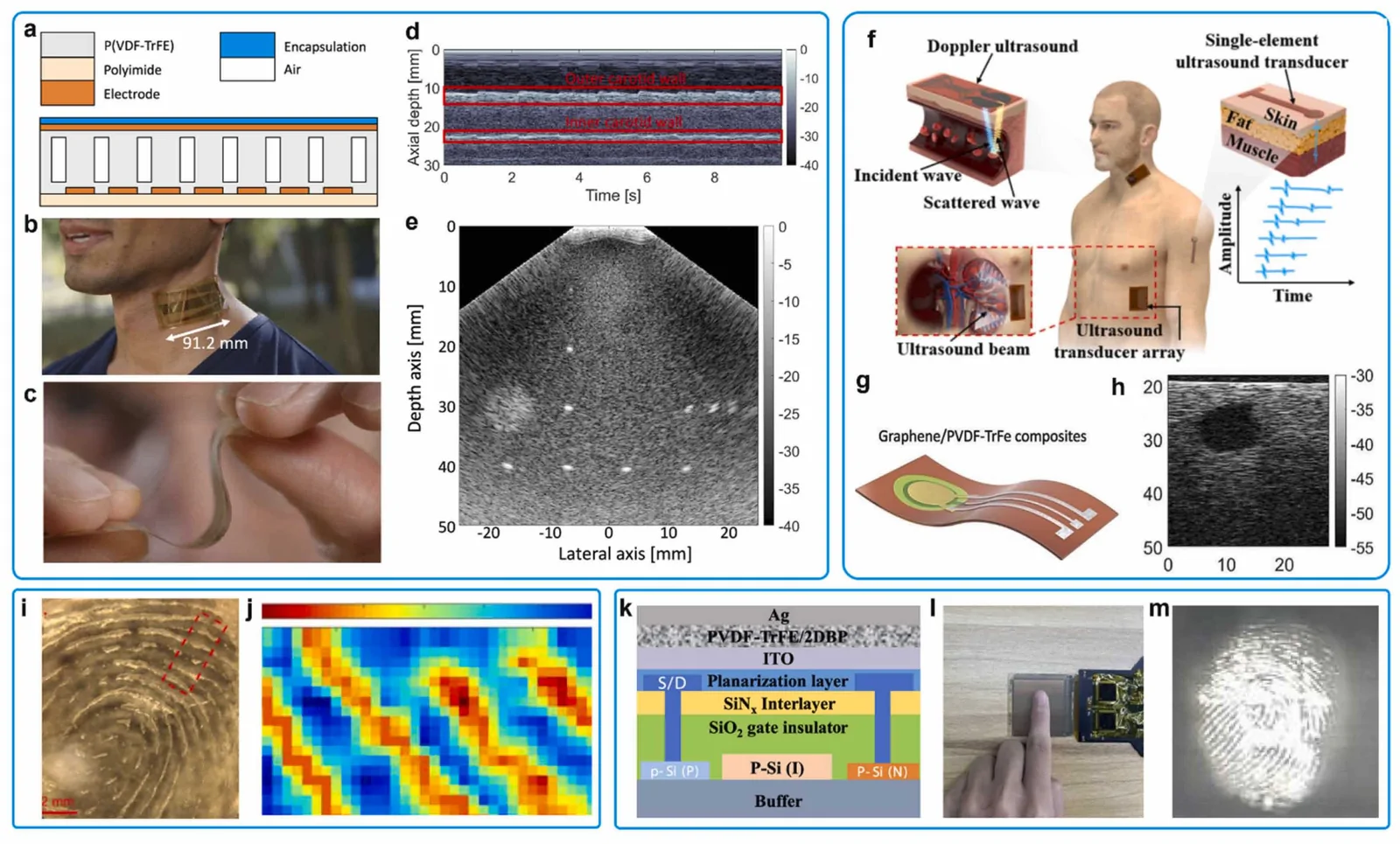
Flexible large-area ultrasound arrays for medical applications [48]: (a) Schematic cross section of the flexible ultrasound transducers embedded with 10μmP(VDF-TrFE) film, (b) photograph of the ultrasound transducer foil, (c) transducer foil placed in the neck on top of the carotid artery for blood pressure measurements, (d) ultrasound data of the carotid of the optimally positioned array element, and (e) imaging of tissue-mimicking phantom. Integration of porous graphene and 3D-printed piezopolymer for flexible ultrasound transducers [49]: (f) Schematic of applications of the flexible transducer (g) Schematic illustration of the layered design, highlighting the main component of the LIG/PVDF-TrFE transducer. (h) B-mode image of the phantom in attenuating Agar. P(VDF TrFE) thin film-based transducer for under display ultrasonic fingerprint sensing applications [51]: (i-j) Electronic image of the fingerprint and the ultrasound imaging of the fingerprint. PVDF−TrFE/2DBP composites for piezoelectric-ultrasonic bimodal electronic skin (PUVE) [50]: (k) thin-film transistor structure unit, (l-m) Pixelation circuit with signal readout circuit and fingerprint pattern recognition.

The primary challenge remains that PVDF exhibits significantly low piezoelectric performance, rendering it ineffective as a transmitter for generating strong pulses. Nonetheless, it retains the potential for entirely soft, skin-integrated ultrasound systems designed for continuous physiological monitoring, rather than imaging. Future directions include material engineering to enhance piezoelectric performance and development of multimodal sensing platforms that combine ultrasound, photoacoustic, and electrophysiological measurements. Advances in scalable fabrication and system integration will enable clinically viable, low-cost devices for applications ranging from maternal-fetal monitoring and neonatal brain imaging to cardiovascular and musculoskeletal health, ultimately transforming ultrasound from a point-of-care modality into a continuous, wearable diagnostic tool.

### 4.2 Photoacoustic and optoacoustic imaging and microscopy

Photoacoustic and optoacoustic imaging combine optical excitation with ultrasonic detection to provide optical absorption contrast at depths beyond those accessible by purely optical imaging [125], [126]. Nanosecond optical excitation induces transient thermoelastic expansion in absorbing chromophores, generating broadband acoustic waves that are detected by ultrasound transducers and reconstructed to visualize tissue structure and function. Endogenous absorbers such as hemoglobin enable label-free visualization of vascular anatomy, blood oxygenation, and hemodynamics, while exogenous contrast agents can further extend molecular and functional imaging capabilities [127], [128], [129]. The broadband nature of PA signals places particular demands on acoustic receivers, making ferroelectric polymers attractive for PA detection.

PVDF and P(VDF-TrFE) possess several characteristics that are particularly favorable for PA/OA receivers, including low acoustic impedance, low mechanical quality factor, broad frequency response, mechanical compliance, and relatively high receiving sensitivity [62]. Compared with conventional piezoelectric ceramics, the acoustic impedance of PVDF-based polymers is substantially closer to that of biological tissue, reducing interfacial reflection and facilitating broadband acoustic coupling. Their low mechanical Q-factor is particularly beneficial for detecting the broadband pressure transients generated in PA imaging, where preservation of both low- and high-frequency components is important for image fidelity and spatial resolution [127], [128], [129], [130]. Although their electromechanical coupling is generally lower than that of high-performance piezoceramics and piezoelectric single crystals, this limitation is less restrictive in receive-dominant PA imaging, making PVDF-based polymers particularly attractive as broadband acoustic detectors [62]. In addition, Thin PVDF and P(VDF-TrFE) films based transparent ultrasound transducers (TUTs) permit light transmission directly through the acoustic aperture, enabling coaxial optical excitation and acoustic detection [62]. This architecture improves optical-acoustic alignment, reduces shadowing, and facilitates compact PA microscopy and tomography systems. Representative devices demonstrate how polymer thickness and transducer geometry can be tailored to different PA imaging regimes (Table 2). Fang *et al.* developed an optically transparent focused PVDF transducer for PA microscopy using transparent electrodes. The device exhibited approximately 60% optical transmittance, a center frequency of 24 MHz, a fractional bandwidth exceeding 100%, and an axial resolution of 14.81 μm [130]. The broad bandwidth and focused geometry enabled high-resolution PA microscopy while allowing optical excitation through the transducer aperture [131].

**Table 2:** Representative performance of ferroelectric polymer-based ultrasound and photoacoustic transducers.

Active materials	Device architecture	Aperture	Thickness (μm)	Freq. (MHz)	−6 dB BW	Sensitivity/output/ insertion loss	Axial/lateral Resolution (µm)	Application	Ref
PVDF	on Esolder	5 × 5	10	39.85	52.88%	-62.03 dB	70.7	Fingerprint imaging	[51]
P(VDF-TrFE)/2DBP	Multi-layers	300	20	-	-	3.2 mV kPa^−1^	50	Fingerprint imaging	[50]
P(VDF-TrFE)	pillar array	91.2 × 14	40	8.2	65%	67 µV/Pa	230	Carotid imaging	[48]
P(VDF-TrFE)/LIG	Multi-layers	3.5	50	8.86	37%	0.044 V kPa^−1^	< 200	Cyst-phantom imaging	[49]
P(VDF-TrFE)/Ni (OH)_2_	In-house	9	26	51.22	102.4%	5.19 V	-	High freq. imaging	[102]
PVDF	on PEI	-	12	48.5	94.2%	-	-	High freq. imaging	[111]
P(VDF-TrFE)	on Si wafer	7.3	11	21.3	143%	IL = 48.5 dB	26/127	High freq. imaging	[132]
P(VDF-TrFE)	on Si wafer	7.5	11	33.2	100%	IL = 60 dB	22	High freq. imaging	[118]
P(VDF-TrFE)	PMUTs	0.8	9	40	92%	-	20/75	IVUS	[52]
PVDF	Circular array	1.5	-	55	80%	-	175	IVUS	[53]
PVDF	In-house	< 5.9	-	32.95	139%	20 dB	26/35	Photoacoustic imaging	[132]
PVDF	Multi-layers	30 × 20	110	6.9	86.3%	-	272.5/400	Photoacoustic imaging	[133]
P(VDF-TrFE)	convex	12	10	24	120%	6.84 µV/Pa	6.6	Photoacoustic imaging	[131]
PVDF	3D wearable	20 × 43	-	9.6	96.4%	-	200	Photoacoustic tomography	[58]
PVDF	Multi-layers	25 × 30	5.5	87	Broadband	-	15/30	Photoacoustic tomography	[134]
PVDF	In-house	12.9	9	24	> 100%	-	14.81	Photoacoustic microscopy	[130]
PVDF	In-house	8	28	13.07	96%	-	194/140	Photoacoustic microscopy	[133]
PVDF	In-house	9	28	27.3	Broadband	-	0.47	Photoacoustic histopathology	[55]
PVDF	Matrix array	1.6 × 1.75	110	10	100%	-	200/1200	Optoacoustic imaging	[57]
P(VDF-TrFE)	In-house	360	20	1–25	Broadband	1.6 μV/Pa	3.73/0.52	Optoacoustic imaging	[135]
P(VDF-TrFE)	In-house	4.6	20	11	Broadband	-	20–30	Optoacoustic angiography	[136]
PVDF	spherical matrix	15.02	-	-	Broadband	22 dB	22–35	Optoacoustic angiography	[56]
P(VDF-TrFE)	spherical	6	20	-	Broadband	6.4 μV/Pa	20	Optoacoustic angiography	[54]

Subsequent P(VDF-TrFE)-based focused transparent transducers further exploited the improved ferroelectric and piezoelectric properties of the copolymer for PA microscopy [133], [137]. Transparent PVDF arrays have further extended this concept from single-element detection toward multichannel PA tomography, while polymer-based spherical and hemispherical arrays have recently demonstrated ultrawideband detection for high-resolution optoacoustic microangiography and tomography [56], [57]. Kim *et al.* developed an ultraviolet-transparent ultrasound transducer (UV-TUT) for reflection-mode ultraviolet photoacoustic microscopy (Fig. 5a-c), achieving a high numerical aperture of 0.38 and a spatial resolution of 0.47 ± 0.03 μm for high-resolution, label-free photoacoustic histopathology [55]. More recently, Perleberg et al. demonstrated a semi-transparent ultrasound transducer for simultaneous PA and laser-induced ultrasound (LUS) imaging, in which a portion of the excitation laser energy is converted into ultrasound while the remaining light is transmitted through the transducer for PA excitation, thereby simplifying the conventional dual-modal imaging architecture and enabling handheld in vivo imaging of animals and humans [138]. Subochev *et al.* developed a 512-element spherical PVDF array comprising sub-mm² elements and exhibiting an ultrawide 0.3–40 MHz detection bandwidth, enabling real-time multiscale volumetric optoacoustic microangiography with 22–35 μm spatial resolution and substantially enhanced signal-to-noise performance compared with piezocomposite counterparts (Fig. 5d-h) [56], [139]. In parallel, Voitovich et al. established a quantitative model relating the noise-equivalent pressure of a calibrated ultrasound detector to the local laser fluence at blood vessels while accounting for acoustic propagation, detector aperture, and bandwidth, providing a useful framework for balancing detector sensitivity, optical exposure, and imaging performance in optical-resolution optoacoustic angiography (Fig. 5i-k) [54]. In summary, these studies demonstrate the evolution of polymer transducers from transparent single-element detectors toward ultrawideband, high-density arrays and integrated multimodal imaging platforms, establishing PVDF and related ferroelectric polymers as promising transducer materials for PA/OA applications ranging from high-resolution microscopy and microangiography to volumetric tomography and future flexible or wearable imaging systems.

**Fig. 5:**
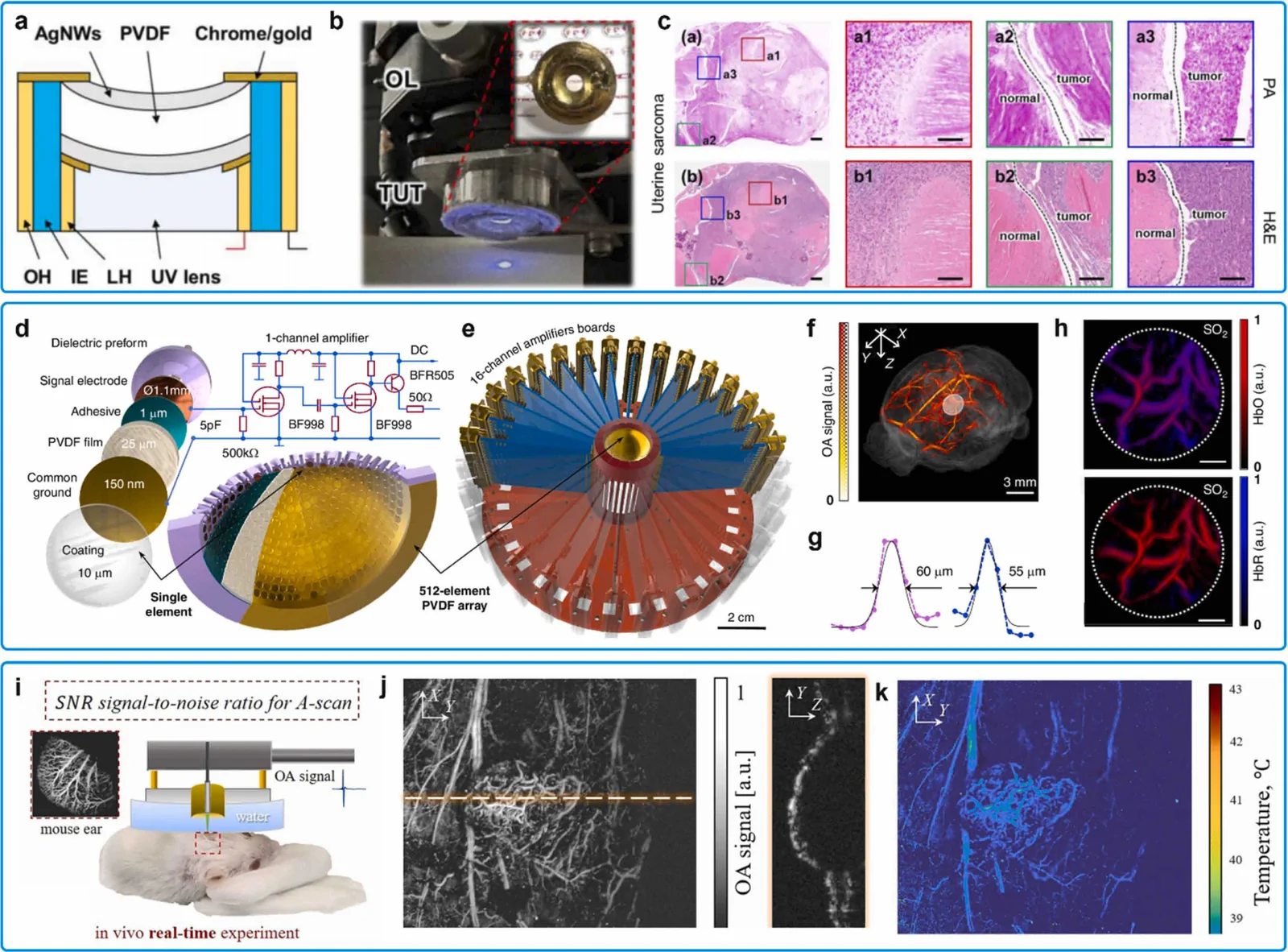
An Ultraviolet-Transparent Ultrasound Transducer Enables High-Resolution Label-Free Photoacoustic Histopathology [55]: (a) A cross-sectional structure of the UV-TUT. (b) Photograph of the UV-TUT, demonstrating its UV transparency. (c) Comparative UV-TUT-PAM and H&E microscopy histology images of murine cancer tissues. Ultrawideband high density polymer-based spherical array for real-time functional optoacoustic micro-angiography: [56] (d) layout of the active detection surface and schematic of the tightly integrated pre-amplification circuitry, (e) Geometrical arrangement of the multichannel electronics, (f) compounded large-scale volumetric OA image of the mouse brain acquired at 850 nm superimposed onto an MRI-based mouse brain atlas, (g) Cerebral vasculature down to 55 µm could be clearly resolved in these transcranial imaging experiments, (h) Snapshots of the representative distributions of the spectrally unmixed HbO and HbR components in selected region. Local laser fluence estimation in optical resolution optoacoustic angiography employing calibrated PVDF ultrasound detector: [54] (i) The schematic of the experimental OR-OAM setup. (j) OAM imaging of the vascular system of tumor with maximum intensity projection along the depth axis and the selected B-scans. (k) the local temperature distribution.

### 4.3 Wearable photoacoustic and optoacoustic imaging

The mechanical flexibility and low acoustic impedance that benefit conventional polymer-based PA receivers are also highly advantageous for wearable photoacoustic imaging, where stable acoustic coupling must be maintained over curved, deformable, and moving biological surfaces. Conventional PA systems generally rely on rigid probes and mechanically fixed optical and acoustic components, limiting continuous measurements during natural motion. Flexible polymer transducers can conform intimately to the skin and other curved surfaces, reducing coupling variations while enabling lightweight and potentially long-term PA acquisition. When combined with flexible optical delivery and miniaturized electronics, these characteristics provide a pathway from conventional handheld PA probes toward skin-integrated functional imaging systems [62], [140].

Earlier studies established physiological and imaging foundations, demonstrating that miniature wearable PA tomography imaging can quantitatively capture hemodynamic and oxygenation changes in biological tissues, particularly in the brain, by leveraging hemoglobin absorption contrast (Fig. 6a-d) [58], [141]. These systems demonstrated that PA imaging could move beyond immobilized measurements and capture dynamic cerebral vascular responses under more physiologically relevant conditions. Although these early systems did not rely primarily on flexible PVDF receiver technology, they established the functional and system-level foundation for subsequent wearable PA devices. More recent developments have incorporated soft and conformal architectures directly into wearable optoacoustic sensing [56], [57], [139], [142]. Liu *et al.* developed a transparent flexible PA transducer based on a 110-μm-thick PVDF film with 150-nm ITO electrodes (Fig. 6f-i) [133]. The device operated at a center frequency of 6.7 MHz with a −3 dB fractional bandwidth of 86.3% and was used for planar, curved-surface, and three-dimensional PA imaging over a reported volume of 20 × 20 × 36 mm³ . Jin *et al.* reported a flexible optoacoustic blood “stethoscope” that integrates optical excitation and acoustic detection in a compact skin-conformal platform (Fig. 6m-o) [57]. The device enables continuous noninvasive monitoring of multiple cardiovascular parameters and demonstrates the potential of wearable optoacoustics for tracking vascular dynamics, oxygenation-related information, and pharmacokinetic processes over time. Compared with conventional snapshot imaging, such continuously coupled devices shift PA technology toward longitudinal physiological monitoring, where conformability, motion tolerance, and stable acoustic coupling become as important as spatial resolution. In addition, the multifunctional flexible photoacoustic device (MFPAD) is highly sensitive to pressure changes, with corresponding light emissions, and efficient sound output modulated by input voltage and frequency (Fig. 6p-r), which enabled real-time posture tracking in robotic fish using an image recognition algorithm based on luminescence [143]. Furthermore, many research focus on engineering innovations in conformal transducers [62], including focused and curved PVDF array [131], [143], 3D PA imaging [144], system integration and signal acquisition strategies [136], [138], and continuous monitoring capabilities compared to conventional rigid probes [145].

**Fig. 6:**
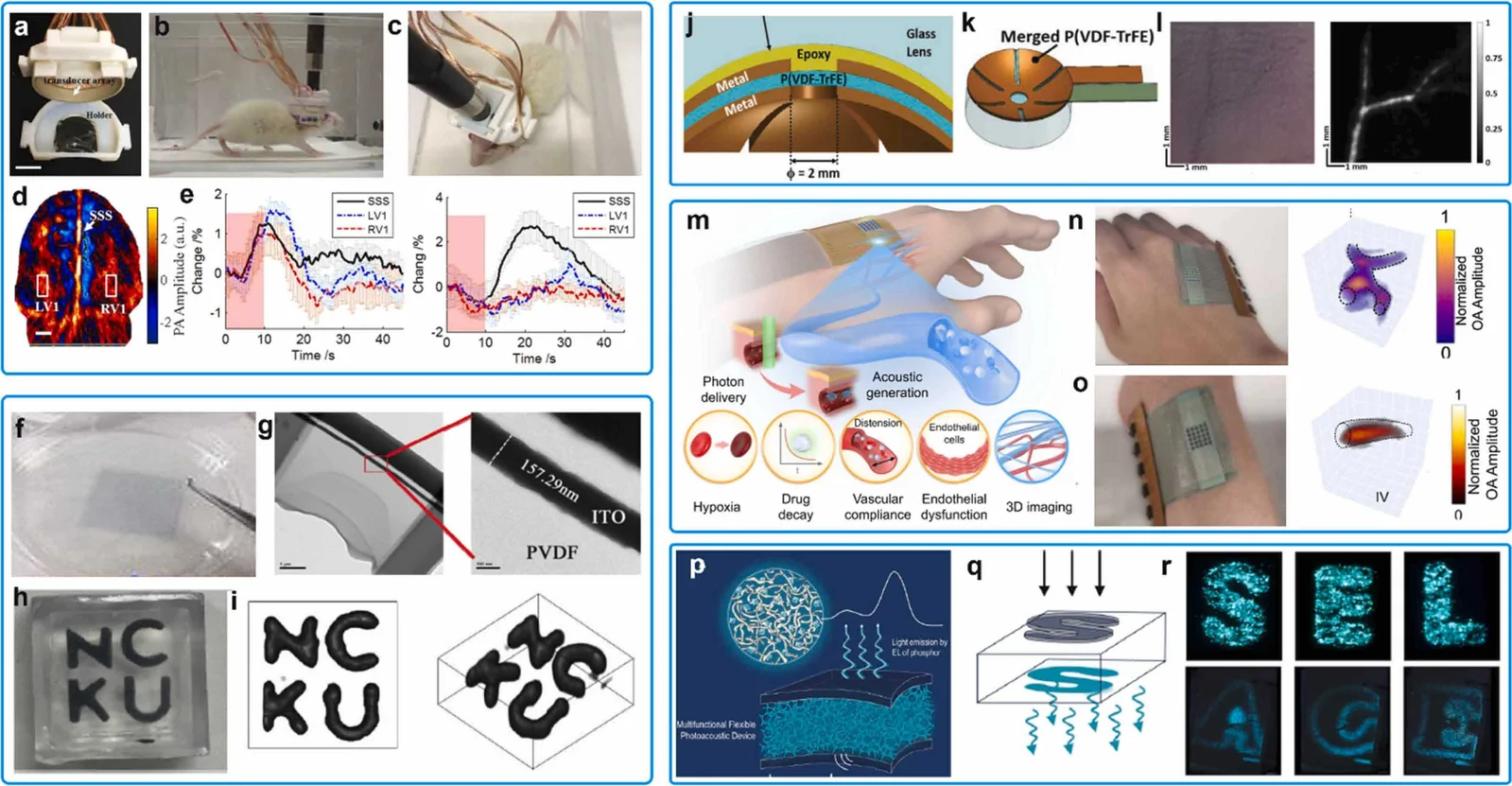
Wearable 3-D photoacoustic tomography for functional brain imaging in behaving rats [58]: (a) Photographs of the fabricated 3D-wPAT prob, (b-c) photographs showing the behaving ability of a rat while wearing 3D-wPAT, (d) The wPAT image from top layer (cortex layer), and (e) Cerebral blood vessel response averaged across the 9 trials. Transparent flexible piezoelectric ultrasound transducer for photoacoustic imaging system [133]: (f) photo of transparent ultrasonic transducer, (g) EM cross section of ITO deposited on PVDF thin film, (h) and (i) Phantom and its imaging results from titled view. An optically transparent focused P(VDF-TrFE) transducer for photoacoustic microscopy [135]: (j) Schematic cross-sectional design of the optically transparent focused P(VDF-TrFE) transducer, (k) P(VDF-TrFE) merged into a spherical shape and bonded onto the concave glass lens, (l) Optical photograph and PAM image of the blood vessels in mouse belly with an area of 8 × 8 mm. Optical photograph and A flexible optoacoustic blood ‘stethoscope’ [57]: (m) Schematic illustration of the OBS laminated on the surface of the hand, (n) Vascular occlusion tests on veins, and (o) Vascular occlusion tests on arteries. PAM image of the blood vessels in mouse belly. Multifunctional flexible photoacoustic device (MFPAD) [143]: (p) Schematic illustration of the structure and working principle of the MFPAD, (q) Schematic illustration of luminescence imaging of spatially patterned constant pressure. (r) Photographs showcasing different types of building blocks placed on the MFPAD.

Several challenges, however, remain before fully wearable polymer-based PA imaging can be achieved. First, the relatively modest electromechanical coupling of PVDF-based polymers compared with piezoceramics can limit detection sensitivity, particularly for weak PA signals originating from deeper tissues. Second, optical transparency, electrical conductivity, and mechanical stretchability must be simultaneously optimized in transparent electrodes and interconnects. Third, wearable systems require stable acoustic coupling during motion, compact optical excitation, multichannel data acquisition, and low-noise electronics without substantially increasing device weight or rigidity [62], [145], [146]. Future wearable PA systems may therefore evolve toward multimodal transparent PA/US patches integrating flexible P(VDF-TrFE) arrays, transparent or stretchable electrodes, compact optical sources, multiplexed electronics, and computational reconstruction within a single conformal platform. Coaxial optical-acoustic architectures are especially attractive because the same sensing aperture could potentially perform pulse-echo ultrasound imaging and broadband PA detection while allowing excitation light to reach the tissue. Integration with AI-assisted reconstruction and spectral analysis could further enable continuous mapping of vascular dynamics and oxygen saturation [147]. Advances in miniaturized fiber-coupled lasers, laser diodes, and other compact optical sources may ultimately reduce the size of the external excitation hardware. Such developments could extend wearable PA/US imaging toward longitudinal cardiovascular monitoring and other applications requiring repeated functional and structural assessment [148].

### 4.4 High- and ultra-high-frequency ultrasound imaging

High- and ultra-high-frequency (UHF) ultrasound imaging is particularly important for visualizing superficial tissues, microscale anatomical structures, and anatomically constrained regions where spatial resolution is more important than penetration depth [149]. High-frequency ultrasound transducers typically operate above approximately 15–20 MHz, while UHF devices can extend to 100 MHz and beyond, enabling spatial resolution from tens of micrometers to the subcellular scale in specialized acoustic microscopy systems [150], [151], [152]. However, frequency-dependent attenuation in biological tissue also increases, limiting penetration depth and creating a fundamental trade-off between imaging resolution and depth. Consequently, high- and UHF ultrasound is particularly suited to ophthalmology, dermatology, intravascular ultrasound (IVUS), small-animal imaging, peripheral tissue imaging, and acoustic microscopy, where the structures of interest are relatively superficial or can be accessed using miniature probes. PVDF-based polymers can be fabricated as uniform films with thicknesses ranging from a few micrometers to tens of micrometers using solution processing, spin coating, printing, film drawing, and related scalable techniques. Their thin-film processability therefore provides a natural route toward high-frequency transducers without the challenging thinning, dicing, and handling processes often required for brittle piezoelectric ceramics and single crystals [153], [154]. Another important advantage of PVDF-based transducers is their inherently broad frequency response. Their relatively low mechanical quality factor allows rapid damping of thickness-mode oscillations and the generation and reception of short acoustic pulses. Fractional bandwidths approaching or exceeding 100% have been demonstrated in appropriately designed polymer transducers [37], [152]. Such broadband behavior is highly desirable for high-resolution pulse-echo imaging because shorter temporal pulses reduce the spatial pulse length and improve axial resolution.

***Ophthalmic imaging and ultrasound biomicroscopy*** represent important biomedical applications of high-frequency PVDF-based transducers [155], [156]. The eye contains numerous small and closely spaced anatomical structures, including the cornea, anterior chamber, iris, ciliary body, lens, retina, and posterior ocular tissues, requiring high spatial resolution for accurate visualization [157]. Conventional diagnostic ophthalmic ultrasound generally uses higher frequencies than abdominal or cardiac imaging, while UBM commonly employs frequencies in the tens-of-megahertz range to resolve structures in the anterior segment. PVDF and P(VDF-TrFE) transducers operating approximately between 20 and 70 MHz have been investigated for these applications [158], [159]. Their broadband response enables short acoustic pulses and improved axial resolution, while the relatively low acoustic impedance facilitates efficient acoustic coupling through water or aqueous coupling media commonly used for ocular imaging. The mechanical flexibility and ease of forming curved or focused polymer films also provide a convenient means of achieving geometric focusing without relying on mechanically complex acoustic lenses. These properties are attractive for resolving fine ocular structures and evaluating conditions involving the anterior chamber, cornea, ciliary body, retina, retinal metabolic rate of oxygen, and other microscale ocular features [138], [160], [161].

***Intravascular ultrasound*** provides another important application in which high frequency and transducer miniaturization are simultaneously required [56], [158]. IVUS transducers must fit within small-diameter catheters while providing sufficient resolution to visualize the vessel wall and atherosclerotic structures [52], [162], [163]. Thin ferroelectric polymer films are advantageous because their high-frequency operation can be achieved without a thick active piezoelectric layer, while their broadband response reduces ringing and improves pulse-echo resolution [164]. Recent developments in P(VDF-TrFE)-based micromachined ultrasonic transducers have further combined polymer piezoelectric films with miniaturized device architectures and integrated electronics. Wang *et al.* developed a miniature focused PVDF-TrFE based PMUT with a 0.8-mm aperture and integrated analog front-end ASIC, exploiting the inherently broad bandwidth (92% at 40 MHz) and approximately 20 μm axial and 75 μm lateral resolution at a 2.4-mm focal depth. It achieved up to 5-mm penetration in rotational IVUS imaging, clearly resolving stent struts and vascular layers, highlighting the potential of broadband PVDF-TrFE transducers for miniaturized, high-resolution IVUS catheters (Fig. 7c-f) [52]. More recently, multimodal intravascular ultrasound and photoacoustic (IVUS/PA) imaging, which utilize dual piezoelectric elements (PVDF and bulk materials), have been widely investigated [165], [166]. López-Marín *et al.* further exploited the favorable acoustic properties of PVDF-TrFE to develop a compact dual-frequency acoustic stack for combined IVUS and intravascular photoacoustic (IVPA) imaging, providing an attractive approach for incorporating high-resolution IVUS and sensitive PA detection within the stringent dimensions of an intravascular catheter [165]. Liu *et al.* investigated beamforming strategies for 55-MHz high-frequency circular-array IVUS, comparing conventional synthetic aperture (SA), apodized synthetic aperture (ASA), and sparse synthetic aperture (SSA) imaging [53], [167]. Notably, ASA improved lateral resolution at 1.5 mm from 260 μm to 175 μm (33%) while tripling the frame rate, whereas SSA reduced the acquired data volume by 50%, demonstrating that optimized beamforming can further improve the resolution–speed tradeoff of high-frequency circular-array IVUS systems.

**Fig. 7:**
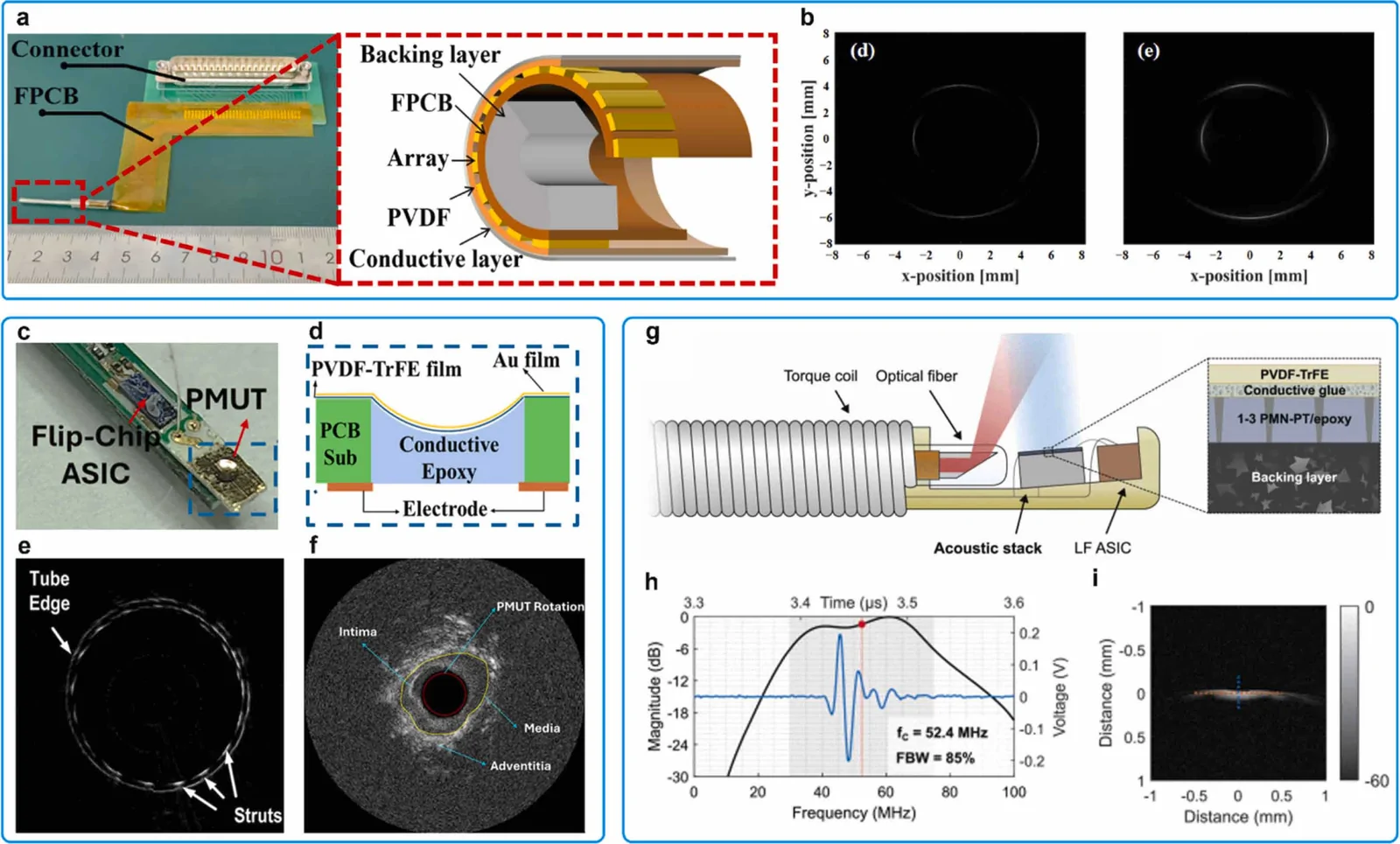
High-frequency circular array imaging algorithms for intravascular ultrasound imaging [53]: (a) Structural diagram of circular array transducer, and (b) Imaging results of scattering phantom using different algorithms. A Miniature, broadband-focused PVDF-TrFE PMUT with interface ASIC for high-resolution IVUS imaging [52]: (c) 1.5-mm-wide portion contained all imaging components to simulate catheter integration, (d) the structure of the PMUT. Rotational ultrasonic image of (e) stent phantom and (f) tissue obtained by recording A-lines while rotating the tower at 1° of rotation per capture. Acoustic stack for combined intravascular ultrasound and photoacoustic imaging [165]: (g) Diagram of the proposed acoustic stack sitting in the tip of a combined IVUS/PA catheter and the top section of the acoustic stack and the interface between its layers. (h) Time and frequency response of the pulse-echo experiment targeting a silver-coated mirror. (i) US imaging of a 15-µm wire at the natural focus of the transducer and 60 dB dynamic range.

PVDF-based materials have also been explored in small-animal imaging, dermatology [168], and acoustic microscopy [169], [170], or superficial tissues such as skin layers, vascular plaques, and peripheral nerves. Future progress in high- and UHF ferroelectric polymer imaging will therefore depend on co-design of the material, acoustic architecture, fabrication process, and electronics, rather than simply increasing operating frequency. Molecular and β-phase engineering may improve intrinsic piezoelectricity, while optimized nanocomposites and multilayer structures may increase sensitivity without excessively increasing acoustic impedance or stiffness. MEMS-compatible fabrication and high-resolution printing could enable increasingly dense flexible arrays, and direct integration with low-noise front-end electronics may mitigate the electrical limitations of miniaturized elements. Together, these developments could extend ferroelectric polymer transducers from conventional single-element high-frequency probes toward flexible, miniaturized, and array-based imaging systems capable of resolving microscale structures in ophthalmology, dermatology, intravascular imaging, small-animal imaging, and acoustic microscopy.

## 5 Challenges and future perspectives

Ferroelectric polymer transducers occupy a distinctive position between conventional rigid piezoelectric materials and emerging soft bioelectronic systems. Their low acoustic impedance, broad bandwidth, mechanical compliance, thin-film processability, and potential optical transparency are particularly advantageous for wearable ultrasound and photoacoustic imaging. However, these benefits are accompanied by fundamental trade-offs among electromechanical efficiency, acoustic output, sensitivity, bandwidth, mechanical flexibility, and long-term stability. No single polymer composition or transducer architecture currently optimizes all of these characteristics simultaneously. Therefore, the future development of ferroelectric polymer ultrasound technologies will depend less on maximizing an individual material parameter and more on co-optimizing materials, acoustic architecture, electronics, mechanics, and imaging algorithms for specific biomedical applications.

A primary challenge remains the relatively modest electromechanical coupling and piezoelectric strain response of conventional PVDF-based polymers compared with high-performance piezoceramics and single crystals. This limitation is particularly important for pulse-echo ultrasound imaging and receiving sensitivity for photoacoustic imaging, where the same transducer must efficiently transmit acoustic energy and detect weak returning echoes. Recent advances in MPB-like ferroelectric polymers, molecular modification, crosslinking, and nanoscale domain engineering are therefore particularly important because they provide opportunities to enhance piezoelectric response without sacrificing the intrinsic mechanical advantages of polymers. Translating these material-level improvements into demonstrable gains in transmit pressure, receive sensitivity, SNR, imaging depth, and long-term reliability should be an important priority for future studies. A second challenge is the transition from individual flexible transducers to high-density imaging arrays. Real-time B-mode, Doppler, volumetric, and beam-steered imaging require arrays with small element pitch, uniform electromechanical properties, dense flexible interconnects, low cross-talk, and scalable multichannel acquisition. Mechanical deformation further changes element positions and orientations, potentially introducing phase errors during beamforming. Future systems will therefore require co-design of conformal array geometry, stretchable interconnects, multiplexing electronics, and deformation-aware beamforming. The acoustic interface represents another critical barrier to continuous wearable imaging. Although the acoustic impedance of PVDF-based materials is closer to that of biological tissue than that of conventional ceramics, stable transmission still depends on the complete transducer–encapsulation–couplant–tissue interface. Motion, curvature, perspiration, dehydration of coupling media, and changes in contact pressure can alter acoustic transmission and degrade image reproducibility during long-term monitoring. Future devices should therefore integrate soft, durable acoustic coupling layers, including hydrogels, organogels, elastomers, or bioadhesive interfaces, directly with the transducer architecture.

For photoacoustic and optoacoustic imaging, polymer transducers offer an additional advantage through their compatibility with optically transparent acoustic apertures. Thin PVDF and P(VDF-TrFE) layers combined with transparent electrodes can enable coaxial optical excitation and acoustic detection, simplifying system alignment and reducing the footprint of multimodal probes. Nevertheless, optical transparency, electrical conductivity, acoustic transmission, flexibility, and fabrication reliability must be simultaneously optimized. Transparent electrodes and encapsulation layers can introduce optical absorption and acoustic attenuation, while high-density transparent arrays require increasingly complex interconnects. Future transparent transducers should therefore be evaluated using combined optical–electrical–acoustic figures of merit rather than optical transmittance or ultrasound sensitivity alone. Integration with compact fiber-coupled sources, laser diodes, and miniaturized acquisition electronics may eventually enable fully wearable PA/US platforms capable of simultaneously mapping anatomy, blood flow, vascular dynamics, and oxygenation.

Overall, the most promising future direction is not simply to replace conventional piezoceramics with softer materials, but to exploit properties that are intrinsic to ferroelectric polymers, including broad bandwidth, low acoustic impedance, mechanical conformability, thin-film manufacturability, and optical transparency, to create imaging architectures that are difficult to realize with rigid transducers. Advances in MPB and molecular engineering may progressively reduce the electromechanical performance gap, while flexible electronics, transparent electrodes, stable acoustic interfaces, and computational imaging can translate these material advances into functional devices. The convergence of these technologies could ultimately enable conformal and transparent ultrasound/photoacoustic systems capable of continuous structural and functional imaging, moving ultrasound from episodic operator-dependent examinations toward longitudinal, wearable, and multimodal biomedical monitoring.

## CRediT authorship contribution statement

**Lin Zhang:** Writing – review & editing, Writing – original draft. **Senyang Xiao:** Writing – review & editing. **Shenghao Wu:** Writing – review & editing. **Jun Xia:** Writing – review & editing. **Qifa Zhou:** Writing – review & editing.

## Declaration of Competing Interest

The authors declare that they have no known competing financial interests or personal relationships that could have appeared to influence the work reported in this paper.

## Data Availability

No data was used for the research described in the article.
